# Evaluation of a novel scoring system based on thrombosis and inflammation for predicting stroke-associated pneumonia: A retrospective cohort study

**DOI:** 10.3389/fnagi.2023.1153770

**Published:** 2023-03-30

**Authors:** Dongze Li, Yi Liu, Yu Jia, Jing Yu, Xiaoli Chen, Hong Li, Lei Ye, Zhi Wan, Zhi Zeng, Yu Cao

**Affiliations:** ^1^Department of Emergency Medicine, West China Hospital, West China School of Medicine, Sichuan University, Chengdu, China; ^2^Laboratory of Emergency Medicine, Disaster Medical Center, West China Hospital, West China School of Medicine, Sichuan University, Chengdu, China; ^3^Department of General Practice, General Practice Medical Center, West China Hospital, West China School of Medicine, Sichuan University, Chengdu, China; ^4^Institute of General Practice, West China Hospital, West China School of Medicine, Sichuan University, Chengdu, China

**Keywords:** inflammation, thrombosis, biomarkers, stroke-associated pneumonia, prognostic score

## Abstract

**Background:**

Inflammation and thrombosis are involved in the development of stroke-associated pneumonia (SAP). Our aim was to evaluate the predictive value of a novel, simplified, thrombo-inflammatory prognostic score (TIPS) that combines both inflammatory and thrombus biomarkers in the early phase of ischemic stroke (IS).

**Methods:**

The study population consisted of 897 patients with a first diagnosis of IS admitted to the emergency department of five tertiary hospitals in China. Of these, the data from 70% of patients was randomly selected to derive the model and the other 30% for model validation. A TIPS of “2” was indicative of high inflammation and thrombosis biomarkers and “1” of one biomarker, with “0” indicative of absence of biomarkers. Multivariate logistic regression analyses were used to identify the association between TIPS and SAP.

**Results:**

The TIPS was an independent predictor of SAP and 90-day mortality, with the incidence of SAP being significantly higher for patients with a high TIPS. The TIPS provided superior predictive value for SAP than clinical scores (A^2^DS^2^) and biomarkers currently used in practice, for both the derivation and validation sets. Mediation analysis revealed that TIPS provided a predictive value than either thrombotic (NLR) and inflammatory (D-dimer) biomarkers alone.

**Conclusion:**

The TIPS score may be a useful tool for early identification of patients at high-risk for SAP after IS.

## Introduction

Ischemic stroke (IS), which accounts for more than 2/3 of all strokes, is a life-threatening emergency associated with high-risk for morbidity and mortality, worldwide (Badve et al., 2019). Among individuals who sustain an acute IS, 7–38% develop stroke-associated pneumonia (SAP), a major adverse clinical complication (Kumar et al., 2010; Nam et al., 2018). SAP is associated with a higher incidence of severe disability, mortality, prolonged hospital stay, and increased financial burden (Katzan et al., 2007; Bruening and Al-Khaled, 2015; Elkind et al., 2020). With regard to prevention of SAP, there has been marked improvement. Specifically, while use of preventive antibiotics was not found to reduce the incidence of SAP (Kalra et al., 2015), despite success in animal studies (Walter et al., 2007; Vermeij et al., 2018), early enteral nutritional support and the use of probiotics have been found to improve immune function and decrease the incidence of stroke-associated infection (Chen et al., 2022). In this regard, a multidisciplinary team approach to improve swallowing function can also lower the risk of SAP occurrence, independent of the severity of IS on admission (Aoki et al., 2016). Consequently, early identification of patients with IS who are at high-risk of SAP and administration of preventive therapy can be crucial in reducing the occurrence of SAP.

Several risk indicators for the development of SAP have been identified: age, dysphagia, atrial fibrillation, stroke severity, and stroke induced immunological suppression (Bruening and Al-Khaled, 2015; Yue et al., 2016; Zhang et al., 2021). Based on these indicators, predictive scoring systems for SAP have been developed, such as the Friedant Pneumonia Predict Score, A^2^DS^2^ Score, Kwon Pneumonia Score, PASS Pneumonia Rule, and ISAN Score (Gong et al., 2016; Ramirez-Moreno et al., 2019; Hotter et al., 2021). These risk indicators and scoring systems, however, reflect the clinical background of patients who are at high-risk for SAP after IS and, thus, are not suitable to assess the risk for SAP. Accordingly, there is a need to develop a novel and reproducible technique to predict for SAP for the rapid identification of patients who are at high-risk for SAP development.

During an IS, activation of the coagulation system and injury to the immune response are main factors of SAP development (Meisel et al., 2005; Zhang et al., 2021). Previous research has shown that some inflammatory markers, including C-reactive protein, procalcitonin (PCT), neutrophil-to-lymphocyte ratio (NLR), and the monocyte-to-lymphocyte ratio, also provide additional predictive information for SAP events in patients with IS (Fluri et al., 2012; Kwan et al., 2013; Salat et al., 2013; Bustamante et al., 2014; Nam et al., 2018; Hotter et al., 2020). In addition, thrombus biomarkers, such as D-dimer and fibrinogen, also serve as SAP predictors (Mele and Turc, 2018; Lin et al., 2022). However, in those studies, the relationship between identified biomarkers and SAP risk in IS was not defined (Hoffmann et al., 2017; Qiu et al., 2022). Therefore, these biomarkers may not fully reflect the severity of different pathological processes involved in SAP due to effects of time-dependent changes and dose–response curves. To date, no single biomarker or dynamic pattern has achieved sufficient accuracy and reliability for clinical application to predict SAP post-IS (Hoffmann et al., 2017; Li et al., 2017b).

Inflammation causes the initiation and spread of coagulation activity, as well as exerting a significant effect on disease conditions, and cardiovascular disease in particular, through the synergistic effects of neutrophils, leukocytes, and platelets (Levi and van der Poll, 2010; Nagareddy and Smyth, 2013). The cellular and molecular link between thrombosis and inflammation supports the thrombus-inflammation state theory, which is related to the severity and complications of IS (Nagareddy and Smyth, 2013). Therefore, a multi-biomarker strategy, which connects the inflammatory state with thrombus biomarkers, could provide incremental predictive information on the risk for SAP, compared to using a single thrombus or inflammatory biomarker.

In previous research, we showed that the thrombo-inflammatory scoring system, which integrates basic thrombus and inflammatory indicators, contributed to the risk classification for aortic dissection, sepsis, and pancreatitis (Li et al., 2015, 2017a, 2018, 2020; Han et al., 2022a,b). In this multicenter retrospective cohort research of patients from the Retrospective Multicenter Study for Ischemic Stroke Evaluation (REMISE) study, we evaluated the hypothesis that a novel thrombo-inflammatory predictive scoring system (TIPS), consisting of a thrombotic and an inflammatory biomarker, could stratify patients with IS on their risk for SAP at the time of admission.

## Materials and methods

### Statement of ethics

Our REMISE study was registered at www.chictr.org.cn (ID: ChiCTR2100052025). The methods of the REMISE study were conducted following the tenets of the Declaration of Helsinki and were approved by the institutional review boards of Sichuan University West China Hospital and other participating hospitals.

### Study population

We designed and validated a novel TIPS system to identify patients at high-risk for SAP after an IS early in the stroke center of emergency department (ED). Our study population included patients with acute IS admitted to the stroke centers of five tertiary hospitals in China, from January 2020 to December 2020. Eligible patients were those with a first diagnosis of IS, according to the 2019 American Heart Association Stroke guideline (Powers et al., 2019). The inclusion criteria were as follows: age > 18 years; first-time diagnosis of IS; and delay from symptom onset to hospitalization of <6 h. The exclusion criteria were: presence of mechanical ventilation within 7 days of the stroke onset; subarachnoid hemorrhage or transient ischemic attack; malignant tumors; severe liver or kidney dysfunction; history of and clinical signs of infection on admission or within 30 days prior to IS onset; and history of surgery within 90 days prior to IS onset.

### Data collection

Demographic and clinical patient data during hospitalization were retrieved from the REMISE study database by trained physicians, using standard case report forms. The following data were extracted: age, sex, vital signs, laboratory test results, body mass index, medical history, arterial blood gas analysis, imaging results, adverse outcomes, and therapies received in the hospital. All laboratory tests and imaging examinations were performed using the standard procedures of Sichuan University West China Hospital.

Stroke-related neurological deficits, at the time of admission, were assessed using the National Institutes of Health Stroke Scale (NIHSS) (Powers et al., 2019), with higher NIHSS scores indicative of more severe neurological impairments. The A^2^DS^2^ score is a validated screening tool for SAP, which includes the predictive factors of age, dysphagia, male sex, atrial fibrillation, and stroke severity, with a higher score being predictive of a higher risk for SAP (Hoffmann et al., 2012). The pneumonia severity index (PSI) is a scoring system to assess the severity of SAP, calculated based on the following factors: age, sex, nursing home residence, comorbidity, physical examination findings, and laboratory and radiographic findings (Aujesky and Fine, 2008).

### Outcome and follow-up

The primary outcome was SAP, diagnosed according to the Centers for Disease Control and Prevention standards of the Outcome Evaluation Committee. The occurrence of SAP was identified based on the electronic medical records of the REMISE study.

### Thrombo-inflammatory prognostic score

The TIPS used in our study was calculated based on inflammatory [WBC, PCT, interleukin-6 (IL-6), C-reaction protein (CRP), neutrophil count, lymphocyte count, and NLR] and thrombotic [PLT, D-dimer, international normalized ratio (INR), activated partial thromboplastin time (APTT), fibrinogen] biomarkers measured at the time of admission. The presence of high inflammation and thrombosis biomarkers was indicative of high-risk for SAP, quantified by a TIPS score of 2. The presence of only one or absence of inflammation or thrombosis biomarkers was assigned a TIPS of “1” and “0,” respectively.

### Statistical analysis

Of the patients included, the data from 70% were randomly used to derive the model (derivation set), with the other 30% used to validate the model (validation set). Classification variables were calculated as a frequency and percentage, with continuous variables calculated as the mean ± standard deviation or median and interquartile range (IQR). An analysis of variance was used to compare parametric patient characteristics and the Kruskal–Wallis H test to compare non-parametric patient characteristics between the SAP and non-SAP groups. The chi-squared (χ^2^) test or Fisher’s exact test was used for between-group comparison of categorical data.

Cut-off values for the different inflammatory (WBC, PCT, IL-6, CRP, neutrophil count, lymphocyte count, and NLR) and thrombotic (PLT, D-dimer, INR, APTT, fibrinogen) biomarkers, used to calculate the TIPS, were evaluated using receiver operating characteristic (ROC) analysis and determined by the maximal corresponding values of Youden’s index. To evaluate the predictive value of TIPS for SAP, the area under the ROC curve (AUC), integrated discrimination improvement (IDI), and net reclassification index (NRI) were calculated for both the derivation and validation sets to identify discrimination (Pencina et al., 2011). Spearman’s correlation analysis was used to determine the relationships between TIPS and other SAP factors. Decision curve analysis was also conducted to evaluate the net benefits of TIPS, decision curve analysis (DCA) was conducted. To explore the indirect effect of TIPS by the A^2^DS^2^ score on SAP, a mediation analysis was performed (Fitzgerald et al., 2015). For all analyses, a two-tailed value of *p* <0.05 was considered statistically significant. All analyses were performed using SPSS Statistics (version 25.0, SPSS, Chicago, IL, United States) and R Studio (version 4.1.3, Vienna, Austria).

## Results

### Baseline characteristics

In total, 897 patients with IS met our criteria for inclusion, with a mean age of 66 ± 13 years and 63.1% of the study sample being men. There were no differences in the demographic and clinical characteristics between patients in the model validation (*n* = 246) and derivation (*n* = 651) sets and the mean A^2^DS^2^ score was 4 (IQR: 2–6) for both the derivation and validation sets. A total of 238 (36.5%) and 81 (32.9%) patients had SAP after IS in the derivation and validation sets, respectively.

### Calculation of TIPS

The AUC for the prediction of SAP using NLR and D-dimer were larger than those for other inflammatory biomarkers (WBC, PCT, IL-6, CRP, neutrophil count, lymphocyte count, and NLR) or thrombotic biomarkers (PLT, D-dimer, INR, APTT, and fibrinogen) in both the derivation and validation sets (Supplementary Table 1). Thus, NLR (cut-off, 3.70) and D-dimer (cut-off, 0.65 mg/L) were used to calculate the optimal TIPS cut-off, based on the maximum Youden’s index (Supplementary Table 2).

The demographic and clinical characteristics of the derivation set according to the TIPS are shown in Table 1. The following patient and clinical variables increased as a function of increasing TIPS: age, WBC, albumin, fibrinogen, cTnT, CK-MB, BNP, and NIHSS score. By contrast, HGB and TG levels decreased with decreasing TIPS. Supplementary Table 3 shows the clinical characteristics according to TIPS in the validation set.

**Table 1:** Relationships between clinical characteristics and the thrombo-inflammatory prognostic score (TIPS) in patients with stroke-associated pneumonia in derivation set.

Variable	TIPS 0 (*n* = 218)	TIPS 1 (*n* = 224)	TIPS 2 (*n* = 209)	*p*-value
Male, *n* (%)	148 (67.90)	142 (63.40)	121 (57.90)	0.101
Age, years	59 ± 12	67 ± 14	69 ± 13	<0.001
Drinking, n (%)	79 (36.20)	60 (26.80)	59 (28.20)	0.069
Smoking, n (%)	106 (48.60)	96 (42.90)	68 (32.50)	0.003
Hypertension, n (%)	129 (59.20)	129 (57.60)	121 (57.90)	0.938
Diabetes, n (%)	65 (29.80)	46 (20.50)	47 (22.50)	0.058
Etiological classification				<0.001
Atherosclerosis, *n* (%)	23 (10.60)	37 (16.50)	33 (15.80)	
Lacunar cerebral infarction, n (%)	45 (20.60)	49 (21.90)	36 (17.20)	
Cardiogenic thrombus, n (%)	18 (8.20)	16 (7.10)	17 (8.10)	
Other, n (%)	15 (6.90)	51 (22.80)	79 (37.80)	
Unknow, n (%)	117 (53.70)	71 (31.70)	44 (21.10)	
WBC, 10^9^/L	6.20 (5.25, 7.38)	7.46 (6.20, 9.58)	8.94 (6.82, 10.90)	<0.001
HGB, 10^9^/L	142 (133, 152)	135 (124, 146)	130 (117, 143)	<0.001
PLT, 10^9^/L	185 (150, 229)	170 (130, 212)	168 (135, 213)	0.021
Fibrinogen, g/L	2.72 (2.34, 3.19)	2.85 (2.37, 3.34)	3.35 (2.57, 4.26)	<0.001
Albumin, g/L	43.3 (40.90, 45.30)	42.1 (39.50, 44.00)	40.4 (37.10, 43.00)	<0.001
BUN, mmol/L	5.20 (4.20, 6.30)	5.80 (4.80, 7.20)	5.90 (4.40, 7.60)	<0.001
Creatinine, μmol/L	71.0 (62.20, 80.80)	74.00 (62.00, 91.00)	73.00 (60.00, 93.00)	0.096
LDL, mmol/L	2.38 (1.90, 3.14)	2.50 (1.90, 3.11)	2.41 (1.85, 2.98)	0.312
HDL, mmol/L	1.15 (0.95, 1.42)	1.22 (0.99, 1.46)	1.21 (0.92, 1.51)	0.249
TG, mmol/L	1.46 (1.02, 2.10)	1.25 (0.97, 1.93)	1.10 (0.80, 1.56)	<0.001
Cys-C, mg/L	0.88 (0.79, 1.02)	0.94 (0.81, 1.12)	0.95 (0.80, 1.15)	0.003
cTnT, pg./mL	8.10 (5.97, 12.2)	11.1 (8.00, 18.10)	15.80 (10.40, 28.30)	<0.001
CK-MB, U/L	1.32 (0.98, 1.88)	1.57 (1.21, 2.150)	1.89 (1.34, 2.84)	<0.001
BNP, pg./mL	82 (39, 210)	333 (80, 904)	825 (276, 1746)	<0.001
NIHSS, score	1.00 (0.00, 4.00)	2.00 (0.00, 10.00)	8.00 (1.00, 15.00)	<0.001
SAP, n (%)	33 (15.10)	66 (29.50)	139 (66.50)	<0.001
A^2^DS^2^, score	3.00 (1.00, 4.00)	4.00 (2.00, 5.00)	5.00 (4.00, 6.00)	<0.001
Therapy, n (%)				0.318
Thrombolysis	44 (20.2)	54 (24.1)	56 (26.8)	
Endovascular treatment	39 (17.9)	31 (13.8)	25 (12.0)	
Only optimal drug treatment	135 (61.9)	139 (62.1)	128 (61.2)	

SBP, systolic blood pressure; DBP, diastolic blood pressure; HGB, hemoglobin; WBC, white blood cell count; PLT, platelet count; BUN, urea nitrogen; LDL, low density lipoprotein; HDL, high density lipoprotein; TG, thyroglobulin; Cys-C, cystatin-C; cTnT cardiac troponins T; CK-MB, creatine kinase-MB; BNP, brain natriuretic peptide; HbA1c, glycated hemoglobin glycosylated hemoglobin; TIPS, thrombo-inflammatory prognostic score; SAP, stroke-associated pneumonia.

### TIPS and SAP risk

The incidence rate of SAP increased as a function of an increasing TIPS. The incidence rate of SAP was 2- to 4-fold higher for patients with a TIPS of 1 or 2, compared to patients with a TIPS of 0. Univariate logistic regression models, shown in Table 2, revealed an association between the TIPS and the incidence of SAP and patient mortality, in both the derivation and validation sets. An increase in TIPS was also independently associated to SAP incidence, after adjusting for latent variables in the multivariate logistic regression analysis, for both the derivation set (TIPS 1 vs. 0: odds ratio (OR): 1.857, 95% confidence interval (95%CI): 1.12–3.081, *p* = 0.016 and TIPS 2 vs. 0: OR: 7.494, 95%CI: 4.402–12.757, *p* < 0.001) and the validation set (TIPS 1 vs. 0: OR: 3.107, 95%CI: 1.139–8.475, *p* = 0.027 and TIPS 2 vs. 0: OR: 13.192, 95%CI: 4.674–37.212, *p* < 0.001) (Table 2).

**Table 2:** Logistic regression analysis regarding correlations between TIPS and clinical outcomes in both the derivation set and the validation set.

Variables	TIPS 1 vs. 0 OR (95% CI)	*p*-value	TIPS 2 vs. 0 OR (95% CI)	*p*-value
Derivation set				
SAP				
Unadjusted	2.342 (1.444–3.742)	<0.001	11.132 (6.967–17.788)	<0.001
Adjusted^a^	1.857 (1.120–3.081)	0.016	7.494 (4.402–12.757)	<0.001
Death				
Unadjusted	2.533 (1.287–4.984)	0.007	4.575 (2.394–8.745)	<0.001
Adjusted^a^	1.871 (0.908–3.855)	0.089	2.757 (1.314–5.783)	0.007
Validation set				
SAP				
Unadjusted	3.696 (1.487–9.189)	0.005	18.089 (7.355–44.489)	<0.001
Adjusted^a^	3.107 (1.139–8.475)	0.027	13.192 (4.674–37.212)	<0.001
Death				
Unadjusted	4.858 (9.609–2.743)	0.016	9.609 (2.743–33.665)	<0.001
Adjusted^a^	4.798 (1.179–19.524)	0.029	2.757 (1.314–5.783)	0.005

^a^Risk factors adjustment included age, sex, drink, smoking, hypertension, diabetes, systolic blood pressure, diastolic blood pressure, pulse, erythrocyte count, hemoglobin, platelet count, international standardized ratio, activated partial thromboplastin time, fibrinogen, creatinine, urea nitrogen and cystatin-C. TIPS, thrombo-inflammatory prognostic score; OR, odds ratio; IC, confidence interval; SAP, stroke-associated pneumonia.

### Comparison between TIPS and other prognostic scores

**Figure 1:**
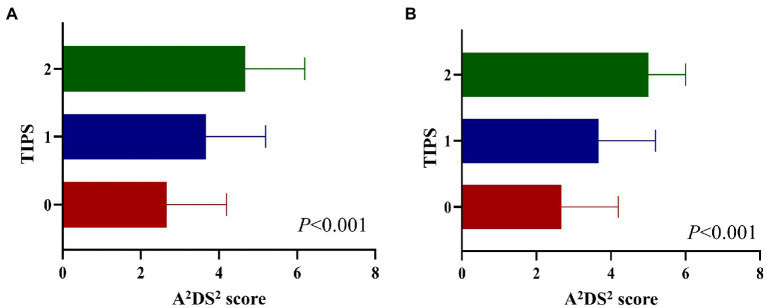
A^2^DS^2^ score in different TIPS scores in the derivation set **(A)**, and validation set **(B)**. A^2^DS^2^, National Institutes of Health Stroke Scale; TIPS, the thrombo-inflammatory prognostic score.

Spearman’s correlation analysis showed that the TIPS was associated with the NIHSS score, PSI, and A^2^DS^2^ (Supplementary Figures 1, 2). Additionally, the A^2^DS^2^ score was higher for patients with a higher TIPS compared to those with a lower TIPS (Figure 1).

### Effect on discrimination and reclassification of TIPS

The AUC for the prediction of SAP using TIPS was 0.762 (95%CI: 0.725–0.802), which was larger than for the A^2^DS^2^ score (0.743, 95%CI: 0.703–0.783) in the derivation set, with similar results for the validation set (Supplementary Table 4).

The IDI value of the TIPS relative to the A^2^DS^2^ score was calculated to confirm the improved discrimination of TIPS (IDI: 0.0293, *p* = 0.027 in the derivation set and 0.0672, *p* = 0.031 in the validation set; Table 3). The potential for clinical reclassification was improved using the TIPS in both the derivation set (NRI: 0.0462, *p* = 0.041) and the validation set (NRI: 0.0148, *p* = 0.047; Table 3). The DCA showed that the net return was higher for TIPS than for the A^2^DS^2^ score at all threshold probabilities in the derivation set (Supplementary Figures 3, 4).

**Table 3:** Reclassification results for stroke-associated pneumonia in both the derivation set and validation set.

	TIPS			
Derivation set^a^				
A^2^DS^2^	≤0.07	0.07–0.29	≥0.29	Reclassified (%)
SAP				
≤0.07	0	0	0	/
0.07–0.29	0	299	67	18
≥0.29	0	99	95	51
Non-SAP				
≤0.07	0	0	0	/
0.07–0.29	0	27	16	37
≥0.29	0	17	31	35
Validation set^b^				
A^2^DS^2^	≤0.07	0.07–0.29	≥0.29	Reclassified (%)
SAP				
≤0.07	0	0	0	/
0.07–0.29	0	39	7	15
≥0.29	0	77	41	65
Non-SAP				
≤0.07	0	0	0	/
0.07–0.29	0	34	9	21
≥0.29	0	16	23	41

^a^NRI (categorical) (95% CI): 0.0462 (0.0854–0.1777); *p*-value: 0.041. IDI (95% CI): 0.0293 (0.0259–0.0845); *p*-value: 0.027. ^b^NRI (categorical) (95% CI): 0.0148 (0.136–0.1656); *p*-value: 0.047. IDI (95% CI): 0.0672 (0.006–0.1284); *p*-value: 0.031. TIPS, thrombo-inflammatory prognostic score; SAP, stroke-associated pneumonia.

### Subgroup analysis

On subgroup analysis to evaluate the effect of sex age, alcohol consumption, smoking, hypertension, diabetes, WBC, PLT, lymphocyte count, neutrophil count, DD, creatinine, and the A^2^DS^2^ score, TIPS remained an independent predictor of SAP (Supplementary Tables 5, 6).

### Mediation analysis

In the mediation analysis, TIPS had a greater effect as a mediator of A^2^DS^2^ and SAP (effect ratio, 28%) than NLR (effect ratio, 14%) and D-dimer (effect ratio, 9%) in the derivation set, with similar results were observed in the validation set (Table 4).

**Table 4:** Direct and indirect effects of D-dimer, NLR and TIPS on A^2^DS^2^ and stroke-associated pneumonia in both the derivation set and validation set.

Variables	Effect	*β* coefficient	Boot 95%CI	Effect ratio (%)	SE	*Z*	*P*
Derivation set							
Thrombus							
D-dimer	Indirect	0.031	(0.014, 0.051)	9	0.010	3.234	<0.001
A^2^DS^2^	Direct	0.323	(0.246, 0.402)	91	0.041	7.908	<0.001
	Total	0.353	(0.273, 0.437)	100	0.041	8.571	<0.001
Inflammation							
NLR	Indirect	0.051	(0.029, 0.075)	14	0.051	4.326	<0.001
A^2^DS^2^	Direct	0.302	(0.221, 0.386)	86	0.041	7.326	<0.001
	Total	0.353	(0.273, 0.437)	100	0.041	8.571	<0.001
Thrombus combined inflammation							
TIPS	Indirect	0.100	(0.067, 0.137)	28	0.018	5.625	<0.001
A^2^DS^2^	Direct	0.253	(0.166, 0.341)	72	0.043	5.834	<0.001
	Total	0.353	(0.273, 0.437)	100	0.041	8.571	<0.001
Validation set							
Thrombus							
D-dimer	Indirect	0.035	(0.014, 0.051)	7	0.020	3.234	0.082
A^2^DS^2^	Direct	0.438	(0.246, 0.402)	93	0.067	7.908	<0.001
	Total	0.473	(0.273, 0.437)	100	0.041	8.571	<0.001
Inflammation							
NLR	Indirect	0.052	(0.008, 0.088)	11	0.020	2.639	0.008
A^2^DS^2^	Direct	0.421	(0.304, 0.551)	89	0.063	6.644	<0.001
	Total	0.473	(0.353, 0.599)	100	0.063	7.564	<0.001
Thrombus combined inflammation							
TIPS	Indirect	0.098	(0.053, 0.183)	21	0.034	2.918	<0.001
A^2^DS^2−^	Direct	0.362	(0.229, 0.495)	79	0.067	5.381	<0.001
	Total	0.460	(0.344, 0.596)	100	0.065	7.098	<0.001

β coefficient was calculated by standard regression equation. TIPS, thrombo-inflammatory prognostic score; A^2^DS^2^, National Institutes of Health Stroke Scale; NLR, neutrophil to lymphocyte ratio; IC, confidence interval.

## Discussion

**Figure 2:**
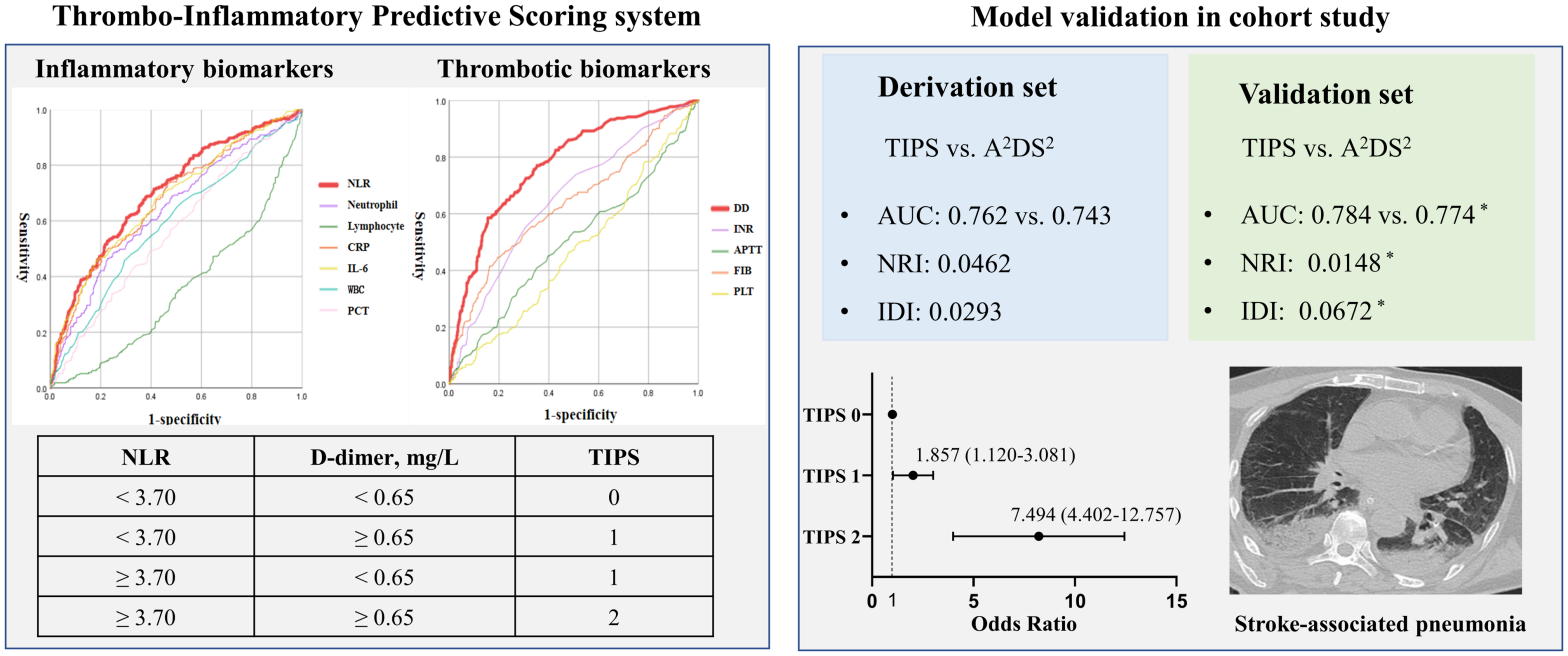
Visual summary: thrombo-inflammatory predictive scoring system (TIPS): early recognizing the risk of stroke-associated pneumonia.

The main finding of our study is that a higher TIPS, based on thrombo-inflammatory biomarkers, was associated with an increased risk and severity of SAP, and an elevated TIPS was identified as an independent predictor of SAP in patients with IS, after adjusting for confounding factors, irrespective of the IS severity. The discrimination and accuracy of the TIPS was superior to that of the A^2^DS^2^ score. Our analyses show than the thrombo-inflammatory status estimated by TIPS may mediate the association between the comprehensive clinical risk score (A^2^DS^2^) and SAP, with the mediating effect of thrombo-inflammatory on SAP being higher than for a single inflammatory or thrombotic state. Consequently, our results support the TIPS as a useful tool for the early identification of high-risk SAP in patients with IS at admission (Figure 2).

SAP is one of the conditions having the greatest impact on the outcome of stroke patients. Stroke induces an inhibition of the immune response, through activation of the autonomic nervous system *via* the stress axis, which significantly promotes the development of SAP (Meisel et al., 2005). Accumulating evidence has shown that biomarkers of thrombosis and inflammation have important value in the early prediction of SAP. Fluri et al. have shown that copeptin, PCT, WBC, and CRP, measured at the time of admission after an IS, are predictors of SAP within 5 days after a stroke, with the combination of biomarkers providing a better predictive accuracy for SAP than each marker independently (*p* = 0.0001) (Fluri et al., 2012). The NLR is higher in patients with SAP and appears to be associated with the severity of SAP (Nam et al., 2018). As important immunological mediators in post-stroke responses, cytokines have been confirmed as independent predictors of SAP, including IL-6, IL-10, and TNFα (Salat et al., 2013; Bustamante et al., 2014; Ashour et al., 2016). As an example, IL-6 is a key biomarker for SAP in the first 2 years after stroke (OR: 19.2; 95%CI: 3.68, 100; *p* < 0.001) (Kwan et al., 2013). The results of one study that pooled the data of two acute stroke trials (STRAWINSKI and PREDICT) showed that PCT and copeptin were independent predictors of SAP at 3 months after a stroke (Hotter et al., 2020). In our study, we identified that some biomarkers of inflammation could provide predictive information for SAP and the power of NLR was superior to that of other biomarkers, such as WBC count, neutrophil count, and lymphocyte count.

Fibrinogen, platelet count, INR, APTT, prothrombin time, and D-dimer in SAP patients were significantly higher than those in non-SAP patients. The fibrinogen-to-albumin ratio (FAR, ≥0.0977) has been reported to be an independent predictor of high-risk of SAP (OR: 2.830; 95%CI: 1.654–4.840), with both the FAR and platelet-to-lymphocyte ratio (PLR) being associated with the severity of SAP (Lin et al., 2022). Therefore, the FAR and PLR show that increased thrombotic state is associated with SAP post-stroke and thus, can be useful to identify patients at high-risk for SAP. Our data showed that D-dimer has a stronger predictive value for SAP than PLT, INR, APTT, and fibrinogen. It is important to consider the extensive interaction between the coagulation and inflammation state.

Inflammation surges immediately after an injury, leading, in the acute phase, to excessive activation of the coagulation system, with the resulting coagulation state greatly increasing the inflammatory process (Levi and van der Poll, 2010). Therefore, the cellular and molecular link between thrombosis and inflammation supports the concept of a thrombus-inflammation state being associated with the severity and complications of IS. Our previous studies have shown that a multi-biomarker strategy, which connects inflammation to thrombus biomarkers, can provide more accurate information about vascular disease and sepsis than information provided by a single thrombus or inflammatory biomarker (Li et al., 2015, 2017a,b, 2018, 2020). Combining the mean platelet volume (MPV)/PC ratio and WBC count could predict in-hospital complications at the time of admission and long-term outcomes in acute type A aortic dissection (Li et al., 2015). A combination of inflammatory (PCT) and thrombotic (DD) biomarkers is useful for risk stratification of adverse clinical outcomes in patients with sepsis (Li et al., 2018). In addition, the thrombo-inflammatory prognostic score (TIPS) we calculated, based on D-dimer and PCT levels, improved the risk stratification of patients with sepsis (Li et al., 2020). In our current study, we built on this evidence, developing a novel TIPS system, which combines thrombo-inflammatory biomarkers (NLR and DD), which provided greater predictive value for SAP. The predictive value of the TIPS was not inferior to the traditional assessment tool (A^2^DS^2^) and single biomarkers of thrombo-inflammation, irrespective of the severity of IS.

The limitations of our study need to be acknowledged. First, this was a single-center retrospective cohort study and, thus, causal effects between the TIPS, its associated thrombo-inflammatory biomarkers, and SAP cannot be inferred. Second, model derivation and validation was based on biomarkers measured in the ED at the time of admission, with changes in these biomarkers, measured at different time points, not considered. As such, the value of TIPS to predict SAP at different time points post-IS was not evaluated. Third, we did not evaluate the predictive value of TIPS in IS patients in some subgroups including different kinds of therapies, and more than 6 h of symptom onset to hospitalization, and so on. Forth, some additional inflammatory thrombus biomarkers were not used in the TIPS model derivation. Lastly, we did not evaluate the time required to complete the TIPS during admission in the ED. Therefore, further multicenter, prospective, studies are needed to confirm the predictive value of TIPS for SAP and its application in practice.

In summary, we provide findings of the predictive value of the TIPS, which is based on thrombo-inflammatory biomarkers for SAP in the early phase of IS onset. We show that an elevated TIPS was an independent predictor of SAP after an IS. In addition, the discrimination and accuracy of SAP were superior to those of the A^2^DS^2^ score. Therefore, the TIPS score is a useful scoring system to identify patients who are at high-risk for SAP after an IS and, as a supplementary tool, it could improve the predictive power of traditional SAP risk scores. Further well-designed studies are required to confirm these results.

## Data availability statement

The data analyzed in this study is subject to the following licenses/restrictions: The data that support the findings of this study are available on request from the corresponding author. Requests to access these datasets should be directed to ZZ, zengzhi2013@qq.com.

## Ethics statement

The studies involving human participants were reviewed and approved by the institutional review boards of Sichuan University West China Hospital. Written informed consent to participate in this study was provided by the participants’ legal guardian/next of kin.

## Author contributions

DL, ZZ, and YC conceived the study design. DL, YJ, JY, YL, XC, HL, and ZW collected the epidemiological and clinical data. DL, YJ, YL, and ZW summarized the data and performed the statistical analyses. DL, YL, and YC interpreted the data and drafted the manuscript. LY and ZZ participated in the design of the study, acquired the data, and helped to revise the manuscript. All authors have accepted responsibility for the entire content of the submitted manuscript and approved its submission.

## Funding

This work was supported the Sichuan Science and Technology Program (Nos. 2022YFS0279 and 2021YFQ0062), Sichuan Provincial Health Commission (No. ZH2022-101), and Sichuan University West China Nursing Discipline Development Special Fund Project (No. HXHL21016).

## Conflict of interest

The authors declare that the research was conducted in the absence of any commercial or financial relationships that could be construed as a potential conflict of interest.

## Publisher’s note

All claims expressed in this article are solely those of the authors and do not necessarily represent those of their affiliated organizations, or those of the publisher, the editors and the reviewers. Any product that may be evaluated in this article, or claim that may be made by its manufacturer, is not guaranteed or endorsed by the publisher.

## Figures and Tables

**Table:** 

APTT	activated partial thromboplastin time
AUC	area under the ROC curve
CRP	C-reaction protein
DCA	decision curve analysis
ED	emergency department
FAR	fibrinogen-to-albumin ratio
IDI	integrated discrimination improvement
INR	international normalized ratio
IQR	interquartile range
IS	ischemic stroke
IL-6	interleukin-6
NIHSS	National Institutes of Health Stroke Scale
NLR	neutrophil-to-lymphocyte ratio
NRI	net reclassification index
OR	odds ratio
PCT	procalcitonin
PLR	platelet-to-lymphocyte ratio
PSI	pneumonia severity index
REMISE	Retrospective Multicenter Study for Ischemic Stroke Evaluation study
ROC	receiver operating characteristic curve
SAP	stroke-associated pneumonia
TIPS	thrombo-inflammatory predictive scoring system

## References

[ref1] Aoki S. Hosomi N. Hirayama J. Nakamori M. Yoshikawa M. Nezu T.. (2016). The multidisciplinary swallowing team approach decreases pneumonia onset in acute stroke patients. PLoS One 11:e0154608. doi: 10.1371/journal.pone.0154608 27138162 PMC4854465

[ref2] Ashour W. Al-Anwar A. D. Kamel A. E. Aidaros M. A. (2016). Predictors of early infection in cerebral ischemic stroke. J. Med. Life 9, 163–169. 27453748 PMC4863508

[ref3] Aujesky D. Fine M. J. (2008). The pneumonia severity index: A decade after the initial derivation and validation. Clin. Infect. Dis. 47, S133–S139. doi: 10.1086/591394, PMID: 18986279

[ref4] Badve M. S. Zhou Z. van de Beek D. Anderson C. S. Hackett M. L. (2019). Frequency of post-stroke pneumonia: Systematic review and meta-analysis of observational studies. Int. J. Stroke 14, 125–136. doi: 10.1177/1747493018806196, PMID: 30346258

[ref5] Bruening T. Al-Khaled M. (2015). Stroke-associated pneumonia in thrombolyzed patients: Incidence and outcome. J. Stroke Cerebrovasc. Dis. 24, 1724–1729. doi: 10.1016/j.jstrokecerebrovasdis.2015.03.045, PMID: 26051666

[ref6] Bustamante A. Sobrino T. Giralt D. García-Berrocoso T. Llombart V. Ugarriza I.. (2014). Prognostic value of blood interleukin-6 in the prediction of functional outcome after stroke: A systematic review and meta-analysis. J. Neuroimmunol. 274, 215–224. doi: 10.1016/j.jneuroim.2014.07.015, PMID: 25091431

[ref7] Chen X. Hu Y. Yuan X. Yang J. Li K. (2022). Effect of early enteral nutrition combined with probiotics in patients with stroke: A meta-analysis of randomized controlled trials. Eur. J. Clin. Nutr. 76, 592–603. doi: 10.1038/s41430-021-00986-3, PMID: 34302128

[ref8] Elkind M. Boehme A. K. Smith C. J. Meisel A. Buckwalter M. S. (2020). Infection as a stroke risk factor and determinant of outcome after stroke. Stroke 51, 3156–3168. doi: 10.1161/STROKEAHA.120.030429, PMID: 32897811 PMC7530056

[ref9] Fitzgerald M. Saville B. R. Lewis R. J. (2015). Decision curve analysis. JAMA 313, 409–410. doi: 10.1001/jama.2015.37 25626037

[ref10] Fluri F. Morgenthaler N. G. Mueller B. Christ-Crain M. Katan M. (2012). Copeptin, procalcitonin and routine inflammatory markers-predictors of infection after stroke. PLoS One 7:e48309. doi: 10.1371/journal.pone.0048309, PMID: 23118979 PMC3485149

[ref11] Gong S. Zhou Z. Zhou M. Lei Z. Guo J. Chen N.. (2016). Validation of risk scoring models for predicting stroke-associated pneumonia in patients with ischaemic stroke. Stroke Vasc. Neurol. 1, 122–126. doi: 10.1136/svn-2016-000025, PMID: 28959473 PMC5435200

[ref12] Han T. Cheng T. Liao Y. He Y. Liu B. Lai Q.. (2022a). Development and validation of a novel prognostic score based on thrombotic and inflammatory biomarkers for predicting 28-day adverse outcomes in patients with acute pancreatitis. J. Inflamm. Res. 15, 395–408. doi: 10.2147/JIR.S344446, PMID: 35068938 PMC8769056

[ref13] Han T. Cheng T. Liao Y. Lai Q. Tang S. Liu B.. (2022b). Thrombo-inflammatory prognostic scores improve BISAP-based risk stratification in acute pancreatitis patients: A retrospective cohort study. J. Inflamm. Res. 15, 3323–3335. doi: 10.2147/JIR.S366246, PMID: 35692952 PMC9176634

[ref14] Hoffmann S. Harms H. Ulm L. Nabavi D. G. Mackert B. M. Schmehl I.. (2017). Stroke-induced immunodepression and dysphagia independently predict stroke-associated pneumonia - the PREDICT study. J. Cereb. Blood Flow Metab. 37, 3671–3682. doi: 10.1177/0271678X16671964, PMID: 27733675 PMC5718319

[ref15] Hoffmann S. Malzahn U. Harms H. Koennecke H. C. Berger K. Kalic M.. (2012). Development of a clinical score (A2DS2) to predict pneumonia in acute ischemic stroke. Stroke 43, 2617–2623. doi: 10.1161/STROKEAHA.112.653055, PMID: 22798325

[ref16] Hotter B. Hoffmann S. Ulm L. Meisel C. Bustamante A. Montaner J.. (2021). External validation of five scores to predict stroke-associated pneumonia and the role of selected blood biomarkers. Stroke 52, 325–330. doi: 10.1161/STROKEAHA.120.031884, PMID: 33280547

[ref17] Hotter B. Hoffmann S. Ulm L. Montaner J. Bustamante A. Meisel C.. (2020). Inflammatory and stress markers predicting pneumonia, outcome, and etiology in patients with stroke. Neurol. Neuroimmunol. Neuroinflamm. 7:e692. doi: 10.1212/NXI.0000000000000692 32098866 PMC7051196

[ref18] Kalra L. Irshad S. Hodsoll J. Simpson M. Gulliford M. Smithard D.. (2015). Prophylactic antibiotics after acute stroke for reducing pneumonia in patients with dysphagia (STROKE-INF): A prospective, cluster-randomised, open-label, masked endpoint, controlled clinical trial. Lancet 386, 1835–1844. doi: 10.1016/S0140-6736(15)00126-9, PMID: 26343840

[ref19] Katzan I. L. Dawson N. V. Thomas C. L. Votruba M. E. Cebul R. D. (2007). The cost of pneumonia after acute stroke. Neurology 68, 1938–1943. doi: 10.1212/01.wnl.0000263187.08969.45 17536051

[ref20] Kumar S. Selim M. H. Caplan L. R. (2010). Medical complications after stroke. Lancet Neurol. 9, 105–118. doi: 10.1016/S1474-4422(09)70266-2 20083041

[ref21] Kwan J. Horsfield G. Bryant T. Gawne-Cain M. Durward G. Byrne C. D.. (2013). IL-6 is a predictive biomarker for stroke associated infection and future mortality in the elderly after an ischemic stroke. Exp. Gerontol. 48, 960–965. doi: 10.1016/j.exger.2013.07.003, PMID: 23872300

[ref22] Levi M. van der Poll T. (2010). Inflammation and coagulation. Crit. Care Med. 38, S26–S34. doi: 10.1097/CCM.0b013e3181c98d21 20083910

[ref23] Li D. Cheng Y. Yu J. Jia Y. Liu B. Xia Y.. (2020). Thrombo-inflammatory prognostic score improves qSOFA for risk stratification in patients with sepsis: A retrospective cohort study. Clin. Chem. Lab. Med. 58, 625–634. doi: 10.1515/cclm-2019-0864, PMID: 31782945

[ref24] Li D. Z. Li X. M. Sun H. P. Yang Y. N. Ma Y. T. Qu Y. Y.. (2015). A novel simplified thrombo-inflammatory prognostic score for predicting in-hospital complications and long-term mortality in patients with type A acute aortic dissection: A prospective cohort study. Eur. Heart J. Suppl. 17, C26–C33. doi: 10.1093/eurheartj/suv032

[ref25] Li D. Ye L. Yu J. Deng L. Liang L. Ma Y.. (2017a). Significance of the thrombo-inflammatory status-based novel prognostic score as a useful predictor for in-hospital mortality of patients with type B acute aortic dissection. Oncotarget 8, 79315–79322. doi: 10.18632/oncotarget.18105, PMID: 29108310 PMC5668043

[ref26] Li D. Yu J. Zeng R. Zhao L. Wan Z. Zeng Z.. (2017b). Neutrophil count is associated with risks of cardiovascular diseases. J. Am. Coll. Cardiol. 70, 911–912. doi: 10.1016/j.jacc.2017.04.070 28797366

[ref27] Li D. Zhou Y. Yu J. Yu H. Xia Y. Zhang L.. (2018). Evaluation of a novel prognostic score based on thrombosis and inflammation in patients with sepsis: A retrospective cohort study. Clin. Chem. Lab. Med. 56, 1182–1192. doi: 10.1515/cclm-2017-0863, PMID: 29794247

[ref28] Lin G. Hu M. Song J. Xu X. Liu H. Qiu L.. (2022). High fibrinogen to albumin ratio: A novel marker for risk of stroke-associated pneumonia? Front. Neurol. 12:747118. doi: 10.3389/fneur.2021.747118 35095715 PMC8792987

[ref29] Meisel C. Schwab J. M. Prass K. Meisel A. Dirnagl U. (2005). Central nervous system injury-induced immune deficiency syndrome. Nat. Rev. Neurosci. 6, 775–786. doi: 10.1038/nrn1765, PMID: 16163382

[ref30] Mele N. Turc G. (2018). Stroke associated with recent mycoplasma pneumoniae infection: A systematic review of clinical features and presumed pathophysiological mechanisms. Front. Neurol. 9:1109. doi: 10.3389/fneur.2018.01109, PMID: 30622505 PMC6308181

[ref31] Nagareddy P. Smyth S. S. (2013). Inflammation and thrombosis in cardiovascular disease. Curr. Opin. Hematol. 20, 457–463. doi: 10.1097/MOH.0b013e328364219d, PMID: 23892572 PMC4086917

[ref32] Nam K. Kim T. J. Lee J. S. Kwon H. Lee Y. Ko S.. (2018). High neutrophil-to-lymphocyte ratio predicts stroke-associated pneumonia. Stroke 49, 1886–1892. doi: 10.1161/STROKEAHA.118.021228 29967014

[ref33] Pencina M. J. D'Agostino R. S. Steyerberg E. W. (2011). Extensions of net reclassification improvement calculations to measure usefulness of new biomarkers. Stat. Med. 30, 11–21. doi: 10.1002/sim.4085, PMID: 21204120 PMC3341973

[ref34] Powers W. J. Rabinstein A. A. Ackerson T. Adeoye O. M. Bambakidis N. C. Becker K.. (2019). Guidelines for the early management of patients with acute ischemic stroke: 2019 update to the 2018 guidelines for the early management of acute ischemic stroke: A guideline for healthcare professionals from the American Heart Association/American Stroke Association. Stroke 50, e344–e418. doi: 10.1161/STR.0000000000000211, PMID: 31662037

[ref35] Qiu H. Song J. Hu J. Wang L. Qiu L. Liu H.. (2022). Low serum transthyretin levels predict stroke-associated pneumonia. Nutr. Metab. Cardiovasc. Dis. 32, 632–640. doi: 10.1016/j.numecd.2021.12.008, PMID: 35105502

[ref36] Ramirez-Moreno J. M. Martinez-Acevedo M. Cordova R. Roa A. M. Constantino A. B. Ceberino D.. (2019). External validation of the A2SD2 and ISAN scales for predicting infectious respiratory complications of ischaemic stroke. Neurologia 34, 14–21. doi: 10.1016/j.nrl.2016.09.003, PMID: 27776955

[ref37] Salat D. Penalba A. García-Berrocoso T. Campos-Martorell M. Flores A. Pagola J.. (2013). Immunological biomarkers improve the accuracy of clinical risk models of infection in the acute phase of ischemic stroke. Cerebrovasc. Dis. 35, 220–227. doi: 10.1159/000346591, PMID: 23466783

[ref38] Vermeij J. D. Westendorp W. F. Dippel D. W. van de Beek D. Nederkoorn P. J. (2018). Antibiotic therapy for preventing infections in people with acute stroke. Cochrane Database Syst. Rev. 2018:D8530. doi: 10.1002/14651858.CD008530.pub3 PMC6491314 29355906

[ref39] Walter U. Knoblich R. Steinhagen V. Donat M. Benecke R. Kloth A. (2007). Predictors of pneumonia in acute stroke patients admitted to a neurological intensive care unit. J. Neurol. 254, 1323–1329. doi: 10.1007/s00415-007-0520-0, PMID: 17361338

[ref40] Yue R. Li D. Yu J. Li S. Ma Y. Huang S.. (2016). Atrial fibrillation is associated with poor outcomes in thrombolyzed patients with acute ischemic stroke: A systematic review and meta-analysis. Medicine 95:e3054. doi: 10.1097/MD.0000000000003054, PMID: 26962831 PMC4998912

[ref41] Zhang S. R. Phan T. G. Sobey C. G. (2021). Targeting the immune system for ischemic stroke. Trends Pharmacol. Sci. 42, 96–105. doi: 10.1016/j.tips.2020.11.010 33341247

