# Supplementary material for: Evaluation of a novel scoring system based on thrombosis and inflammation for predicting stroke-associated pneumonia: A retrospective cohort study
Source: Front Aging Neurosci. 2023 Mar 30;15:1153770. doi: 10.3389/fnagi.2023.1153770 (PMC10098085; doi:10.3389/fnagi.2023.1153770)
Supplement: Supplementary file 1 [file Data_Sheet_1.docx]

**Appendix**

**Supplementary Table 1.** The receiver operating characteristic curve (AUC) for evaluating the stroke-associated pneumonia of inflammatory and thrombotic biomarkers in the derivation and validation set.

**Supplementary Table 2.** The method of calculating the thrombo-inflammatory prognostic score (TIPS) using NLR and D-dimer.

**Supplementary Table 3.** Relationships between clinical characteristics and the thrombo-inflammatory prognostic score in patients with stroke-associated pneumonia in the validation set.

**Supplementary Table 4.** The receiver operating characteristic curve (AUC) for evaluating the stroke-associated pneumonia of A^2^DS^2^ and TIPS in both the derivation set and the validation set.

**Supplementary Table 5.** Subgroup analysis of the association between TIPS and stroke-associated pneumonia by multivariate logistic regression analysis in both the derivation set and the validation set.

**Supplementary Table 6.** Subgroup analysis of the association between TIPS and stroke-associated pneumonia by multivariate logistic regression analysis in the validation set.

**Supplementary Figure 1.** Correlation analysis of A^2^DS^2^, NIHSS, mRS and TIPS in the validation set.

**Supplementary Figure 2.** Correlation analysis of A^2^DS^2^ , NIHSS, mRS and TIPS in the derivation set.

**Supplementary Figure 3.** Decision curve analysis for the outcome of stroke-associated pneumonia in the derivation set.

**Supplementary Figure 4.** Decision curve analysis for the outcome of stroke-associated pneumonia in the validation set.

Supplementary Table 1. The receiver operating characteristic curve (AUC) for evaluating the stroke-associated pneumonia of inflammatory and thrombotic biomarkers in the derivation and validation set.

| **Variables** | **The derivation set** |  |  | **The validation set** |  |
| --- | --- | --- | --- | --- | --- |
|  | **AUC (95% CI)** | ***P***-Value |  | **AUC (95% CI)** | ***P***-Value |
| Inflammatory biomarkers |  |  |  |  |  |
| NLR | 0.719(0.678-0.760) | <0.001 |  | 0.722(0.654-0.790) | <0.001 |
| WBC | 0.663(0.619-0.707) | <0.001 |  | 0.646(0.568-0.723) | <0.001 |
| Neutrophil | 0.699(0.656-0.741) | <0.001 |  | 0.691(0.618-0.764) | <0.001 |
| Lymphocyte | 0.348(0.304-0.392) | <0.001 |  | 0.322(0.251-0.393) | <0.001 |
| PCT | 0.661(0.533-0.788) | <0.001 |  | 0.641(0.478-0.771) | <0.001 |
| IL-6 | 0.691(0.562-0.819) | <0.001 |  | 0.662(0.582-0.810) | <0.001 |
| CRP | 0.709(0.624-0.810) | <0.001 |  | 0.711(0.657-0.814) | <0.001 |
| Thrombotic biomarkers |  |  |  |  |  |
| PLT | 0.468(0.422-0.514) | 0.170 |  | 0.512(0.434-0.590) | 0.768 |
| D-dimer | 0.752(0.713-0.791) | <0.001 |  | 0.777(0.715-0.839) | <0.001 |
| INR | 0.634(0.590-0.679) | <0.001 |  | 0.644(0.569-0.719) | <0.001 |
| APTT | 0.500(0.452-0.547) | 0.987 |  | 0.468(0.388-0.549) | 0.421 |
| Fibrinogen | 0.690(0.629-0.752) | <0.001 |  | 0.617(0.537-0.697) | 0.003 |

NLR, neutrophil-to-lymphocyte Ratio; PCT, procalcitonin; IL-6, interleukin-6; CRP, C-reaction protein; WBC, white blood cell; PLT, platelet; INR, international normalized ratio; APTT, activated partial thromboplastin time.

Supplementary Table 2. The method of calculating the thrombo-inflammatory prognostic score (TIPS) using NLR and D-dimer.

| NLR | D-dimer, mg/L | TIPS |
| --- | --- | --- |
| < 3.70 | < 0.65 | 0 |
| < 3.70 | ≥ 0.65 | 1 |
| ≥ 3.70 | < 0.65 | 1 |
| ≥ 3.70 | ≥ 0.65 | 2 |

NLR, neutrophil-to-lymphocyte Ratio; TIPS, thrombo-inflammatory prognostic score.

Supplementary Table 3. Relationships between clinical characteristics and the thrombo-inflammatory prognostic score in patients with stroke-associated pneumonia in the validation set.

| **Variable** | **TIPS 0 (n=79)** | **TIPS 1 (n=87)** | **TIPS 2 (**n=80) | ***P***-Value |
| --- | --- | --- | --- | --- |
| Male, n (%) | 57 (72.20) | 59 (67.80) | 48 (60.00) | 0.256 |
| Age, years | 58 ± 11 | 65 ± 13 | 68 ± 13 | <0.001 |
| Drink, n (%) | 32 (40.50) | 27 (31.00) | 27 (33.80) | 0.425 |
| Smoking, n (%) | 39 (49.40) | 39 (44.80) | 32 (40.00) | 0.494 |
| Hypertension, n (%) | 45 (57.00) | 48 (55.20) | 49 (61.30) | 0.719 |
| Diabetes, n (%) | 31 (39.20) | 15 (17.20) | 16 (20.00) | 0.002 |
| Etiological classification |  |  |  | <0.001 |
| Atherosclerosisn, n (%) | 11 (13.90) | 16 (18.40) | 13 (16.20) |  |
| Lacunar cerebral infarction, n (%) | 5 (6.33) | 15 (17.20) | 16 (20.00) |  |
| Cardiogenic thrombus, n(%) | 4 (5.06) | 19 (21.80) | 28 (35.00) |  |
| Other, n (%) | 49 (62.00) | 28 (32.20) | 12 (15.00) |  |
| Unknow, n (%) | 10 (12.70) | 9 (10.30) | 11 (13.80) |  |
| WBC, 109/L | 6.04 (5.04, 6.94) | 7.58 (6.28, 10.10) | 8.89 (6.67, 11.00) | <0.001 |
| HGB, 109/L | 144 ± 17 | 137 ± 17 | 131 ± 19 | <0.001 |
| PLT, 109/L | 179 (131, 220) | 173 (132, 229) | 175 (138, 223) | 0.953 |
| Fibrinogen, g/L | 2.72 ± 0.67 | 2.97 ± 0.86 | 3.61 ± 1.44 | <0.001 |
| Albumin, g/L | 43.30 (41.00, 44.70) | 42.80 (40.00, 44.70) | 40.10 (36.20, 42.50) | <0.001 |
| BUN, mmol/L | 5.40 (4.20, 6.40) | 5.50 (4.80, 7.00) | 5.65 (4.32, 7.50) | 0.404 |
| Creatinine, μmol/L | 72.00 (65.50, 80.00) | 74.00 (64.50, 87.50) | 72.00 (58.80, 88.50) | 0.501 |
| LDL, mmol/L | 2.36 (1.82, 3.12) | 2.46 (1.79, 3.14) | 2.46 (1.83, 2.97) | 0.718 |
| HDL, mmol/L | 1.10 (0.91, 1.38) | 1.21 (1.02, 1.46) | 1.23 (0.92, 1.51) | 0.169 |
| TG, mmol/L | 1.47 (1.04, 2.27) | 1.39 (0.98, 1.89) | 1.15 (0.78, 1.72) | 0.017 |
| Cys-C, mg/L | 0.88 (0.80, 0.99) | 0.94 (0.82, 1.11) | 0.92 (0.78, 1.15) | 0.135 |
| cTnT, pg/mL | 7.10 (5.10, 9.27) | 10.6 (7.90, 15.90) | 14.5 (9.25, 26.70) | <0.001 |
| CK-MB, U/L | 1.27 (1.01, 1.74) | 1.59 (1.19, 2.22) | 1.86 (1.41, 3.26) | <0.001 |
| BNP, pg/mL | 76 (33, 116) | 350 (95, 872) | 818 (253, 1576) | <0.001 |
| NIHSS, score | 1.00 (0.00, 3.00) | 2.00 (0.00, 9.00) | 8.00 (1.00, 14.00) | <0.001 |
| SAP, n(%) | 7 (8.86) | 23 (26.40) | 51 (63.70) | <0.001 |
| A2DS2 , score | 3.00 (1.00, 4.00) | 4.00 (2.00, 5.00) | 5.00 (4.00, 6.00) | <0.001 |

SBP, systolic blood pressure; DBP, diastolic blood pressure; HGB, hemoglobin; WBC, white blood cell; PLT, platelet; BUN, urea nitrogen; LDL, low density lipoprotein; HDL, high density lipoprotein; TG, thyroglobulin; Cys-C, cystatin-C; cTnT Troponin T; CK-MB, creatine kinase, MB form; BNP, brain natriuretic peptide; HbA1c,glycated hemoglobin glycosylated hemoglobin; TIPS, thrombo-inflammatory prognostic score; SAP,stroke-associated pneumonia.

Supplementary Table 4. The receiver operating characteristic curve (AUC) for evaluating the stroke-associated pneumonia of A^2^DS^2^ and TIPS in both the derivation set and the validation set.

| **Variables** | **AUC (95% CI)** | ***P*-Value** | **∆AUC** | ***P*-Value** |
| --- | --- | --- | --- | --- |
| Derivation set |  |  | 0.019 | 0.985 |
| A^2^DS^2^ | 0.743(0.703-0.783) | <0.001 |  |  |
| TIPS | 0.762(0.725-0.802) | <0.001 |  |  |
| Validation set |  |  | 0.010 | 0.969 |
| A^2^DS^2^ | 0.774(0.708-0.803) | <0.001 |  |  |
| TIPS | 0.784(0.724-0.844) | <0.001 |  |  |

TIPS, thrombo-inflammatory prognostic score; AUC, area under the receiver-operating characteristic curve; IC, confidence interval.

Supplementary Table 5. Subgroup analysis of the association between TIPS and stroke-associated pneumonia by multivariate logistic regression analysis in both the derivation set and the validation set .

|  | **TIPS 1 vs 0 odds ratio (95% CI)** | ***P*-Value** | **TIPS 2 vs 0 odds ratio (95% CI)** | ***P*-Value** | ***P* for interaction** |
| --- | --- | --- | --- | --- | --- |
| Gender |  |  |  |  | 0.746 |
| male | 2.397 (1.366 4.205) | 0.002 | 3.359(2.511-4.493) | <0.001 |  |
| female | 2.333 (0.990-5.502) | 0.053 | 3.444(2.281-5.199) | <0.001 |  |
| Age |  |  |  |  | 0.484 |
| < 40 | 3.000(0.232-38.875) | 0.401 | 4.000(1.129-14.175) | 0.032 |  |
| 40-60 | 1.769(0.721-4.340) | 0.213 | 4.043(2.541-6.434) | <0.001 |  |
| > 60 | 2.229(1.241-4.004) | 0.009 | 2.960(2.212-3.959) | <0.001 |  |
| Drinking |  |  |  |  | 0.752 |
| No | 2.201(1.249-3.877) | 0.006 | 10.324(5.855-18.201) | <0.001 |  |
| Yes | 2.649(1.140-6.155) | 0.023 | 13.014(4.624-30.116) | <0.001 |  |
| Smoking |  |  |  |  | 0.732 |
| No | 2.187(1.148-4.164) | 0.017 | 10.171(5.467-18.922) | <0.001 |  |
| Yes | 2.557(1.289-5.071) | 0.007 | 13.5(6.410-28.434) | <0.001 |  |
| Hypetension |  |  |  |  | 0.197 |
| No | 2.321(1.110-4.856) | 0.025 | 15.59(7.346-33.083) | <0.001 |  |
| Yes | 2.362(1.287-4.334) | 0.006 | 8.886(4.868-16.219) | <0.001 |  |
| Diabetes |  |  |  |  | 0.471 |
| No | 2.670(1.508-4.729) | 0.001 | 12.587(4.787-18.639) | <0.001 |  |
| Yes | 1.935(0.814-4.600) | 0.135 | 9.429(3.943-22.547) | <0.001 |  |
| WBC, 10^9^/L |  |  |  |  | 0.084 |
| ≤  10 | 2.004(1.214-3.308) | 0.007 | 2.890(2.242-3.725) | <0.001 |  |
| > 10 | 6.667(0.784-56.685) | 0.082 | 6.055(2.084-17.596) | 0.001 |  |
| PLT, 10^9^/L |  |  |  |  | 0.138 |
| ≤  100 | 2.944(1.337-11.204) | 0.457 | 8.062(2.266-28.681) | 0.001 |  |
| > 100 | 2.379(1.460-3.877) | 0.001 | 3.211(2.523-4.085) | <0.001 |  |
| Lymphocyte, 10^9^/L |  |  |  |  | 0.032 |
| ≤  1.21 | 0.57(0.186-1.751) | 0.326 | 5.047(1.852-13.752) | 0.002 |  |
| > 1.21 | 3.304(1.955-5.584) | <0.001 | 11.852(5.799-24.222) | <0.001 |  |
| Neutrophil, 10^9^/L |  |  |  |  | 0.646 |
| ≤ 6.3 | 1.923(1.137-3.253) | 0.015 | 8.560(4.644-15.779) | <0.001 |  |
| > 6.3 | 7.163(0.888-57.769) | 0.065 | 29.023(3.679-228.972) | 0.001 |  |
| D-Dimer, mg/L |  |  |  |  | 0.837 |
| ≤  0.5 | 1.546(0.815-2.955) | 0.182 | 6.477(1.654-16.876) | 0.021 |  |
| > 0.5 | 3.307(0.931-11.750) | 0.064 | 12.576(3.599-43.942) | <0.001 |  |
| Creatinine, μmol/L |  |  |  |  | 0.439 |
| ≤  70 | 2.064(1.028-4.143) | 0.042 | 8.919(4.585-17.352) | <0.001 |  |
| > 70 | 2.643(1.390-5.028) | 0.003 | 13.65(7.041-26.464) | <0.001 |  |
| A^2^DS^2^ |  |  |  |  | 0.006 |
| ≤ 4 | 1.650(0.892-3.055) | 0.111 | 1.991(0.861-4.608) | 0.108 |  |
| > 4 | 2.645(1.747-4.004) | <0.001 | 2.746(2.015-3.743) | <0.001 |  |

TIPS, thrombo-inflammatory prognostic score; mRS, modified rankin scale; WBC, white blood cell; PLT, platelet; OR, odds ratio; IC, confidence interva**l**.

Supplementary Table 6. Subgroup analysis of the association between TIPS and stroke-associated pneumonia by multivariate logistic regression analysis in the validation set .

|  | **TIPS 1 vs 0 odds ratio (95% CI)** | ***P*-Value** | **TIPS 2 vs 0 odds ratio (95% CI)** | ***P*-Value** | ***P* for interaction** |
| --- | --- | --- | --- | --- | --- |
| Gender |  |  |  |  | 0.253 |
| male | 5.363(1.678-17.142) | 0.005 | 9.105(3.908-35.386) | <0.001 |  |
| female | 1.727(0.379-7.864) | 0.48 | 8.143(2.001-33.144) | 0.003 |  |
| Age, years |  |  |  |  | 0.254 |
| < 40 | 3.333(0.204-15.456) | 0.398 | 11.126(2.279-51.178) | 0.046 |  |
| 40-60 | 3.647(0.808-16.457) | 0.092 | 19.190(4.284-85.968) | <0.001 |  |
| > 60 | 4.091(1.098-15.246) | 0.036 | 19.636(5.396-71.457) | <0.001 |  |
| Drink |  |  |  |  | 0.527 |
| No | 3.583(1.102-11.655) | 0.034 | 10.903(6.477-28.463) | <0.001 |  |
| Yes | 4.07(1.098-15.246) | 0.057 | 14.061(5.396-31.457) | <0.001 |  |
| Smoking |  |  |  |  | 0.516 |
| No | 3.000(0.884-10.184) | 0.078 | 13.737(4.204-44.881) | <0.001 |  |
| Yes | 4.714(1.199-18.530) | 0.026 | 16.400(6.543-46.521) | <0.001 |  |
| Hypetension |  |  |  |  | 0.025 |
| No | 4.667(1.792-12.063) | 0.012 | 18.59(13.025-81.329) | <0.001 |  |
| Yes | 1.932(0.649-5.757) | 0.237 | 7.977(2.855-22.288) | <0.001 |  |
| Diabetes |  |  |  |  | 0.854 |
| No | 4.231(1.345-13.310) | 0.014 | 8.333(5.853-17.429) | <0.001 |  |
| Yes | 2.333(0.411-13.258) | 0.339 | 10.533(4.178-20.915) | <0.001 |  |
| WBC, 10^9^/L |  |  |  |  | 0.48 |
| ≤  10 | 3.333(1.275-8.716) | 0.014 | 4.762(3.568-19.933) | <0.001 |  |
| > 10 | 6.912(1.704-60.146) | 0.092 | 7.118(4.561-27.768) | 0.011 |  |
| PLT, 10^9^/L |  |  |  |  | 0.218 |
| ≤  100 | 2.167(1.098-12.097) | 0.312 | 9.121(3.467-19.044) | <0.001 |  |
| > 100 | 3.054(1.195-7.804) | <0.001 | 14.464(5.809-36.017) | <0.001 |  |
| Lymphocyte, 10^9^/L |  |  |  |  | 0.958 |
| ≤  1.21 | 0.196(0.023-1.690) | 0.138 | 2.562(0.399-26.440) | 0.321 |  |
| > 1.21 | 6.732(2.348-19.303) |  | 27.600(6.766-112.58) | <0.001 |  |
| Neutrophil, 10^9^/L |  |  |  |  |  |
| ≤ 6.3 | 2.223(1.087-6.723) | 0.031 | 8.963(4.054-29.455) | 0.003 | 0.947 |
| > 6.3 | 3.209(1.148-16.350) | 0.02 | 14.786(5.088-42.976) | <0.001 |  |
| D-Dimer, mg/L |  |  |  |  | 0.331 |
| ≤  0.5 | 1.813(0.528-6.229) | 0.345 | 5.729(2.228-21.053) | 0.221 |  |
| > 0.5 | 4.107(1.132-14.850) | 0.077 | 13.635(3.601-43.871) | 0.001 |  |
| Creatinine, μmol/L |  |  |  |  | 0.332 |
| ≤  70 | 3.37(0.938-12.105) | 0.063 | 12.732(3.702-43.786) | <0.001 |  |
| > 70 | 4.333(1.148-16.350) | 0.03 | 25.511(6.751-56.407) | <0.001 |  |
| A^2^DS^2^ |  |  |  |  | 0.001 |
| ≤ 4 | 1.72(0.776-8.537) | 0.118 | 1.800(0.734-9.073) | <0.001 |  |
| > 4 | 2.429(0.270-7.549) | <0.001 | 3.991(0.759-19.933) | <0.001 |  |

TIPS, thrombo-inflammatory prognostic score; WBC, white blood cell; PLT, platelet; OR, odds ratio; IC, confidence interval.


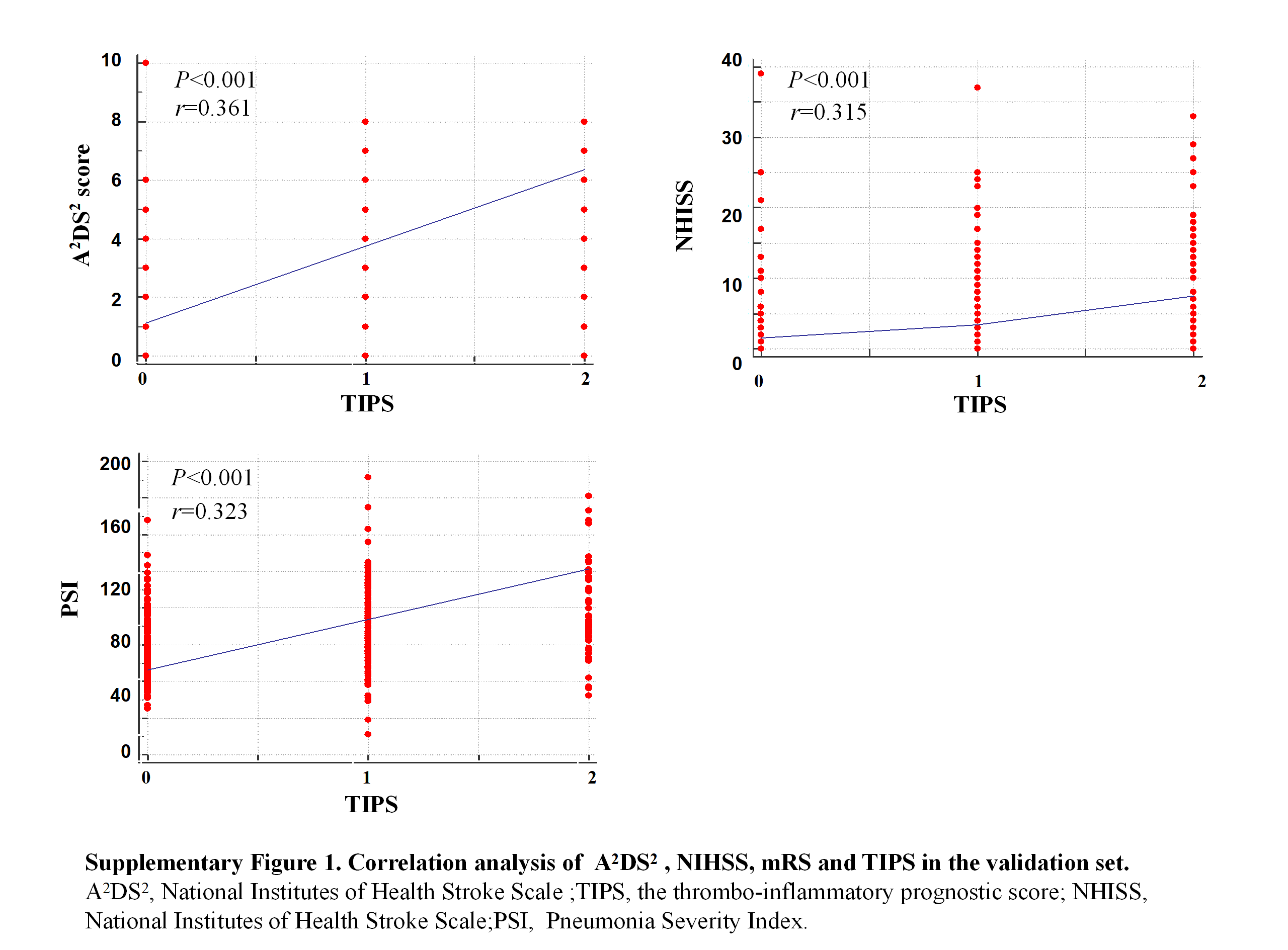


Supplementary Figure 1. Correlation analysis of A^2^DS^2^ , NIHSS, mRS and TIPS in the validation set.

A^2^DS^2^, National Institutes of Health Stroke Scale ;TIPS, the thrombo-inflammatory prognostic score; NHISS, National Institutes of Health Stroke Scale;PSI, Pneumonia Severity Index.


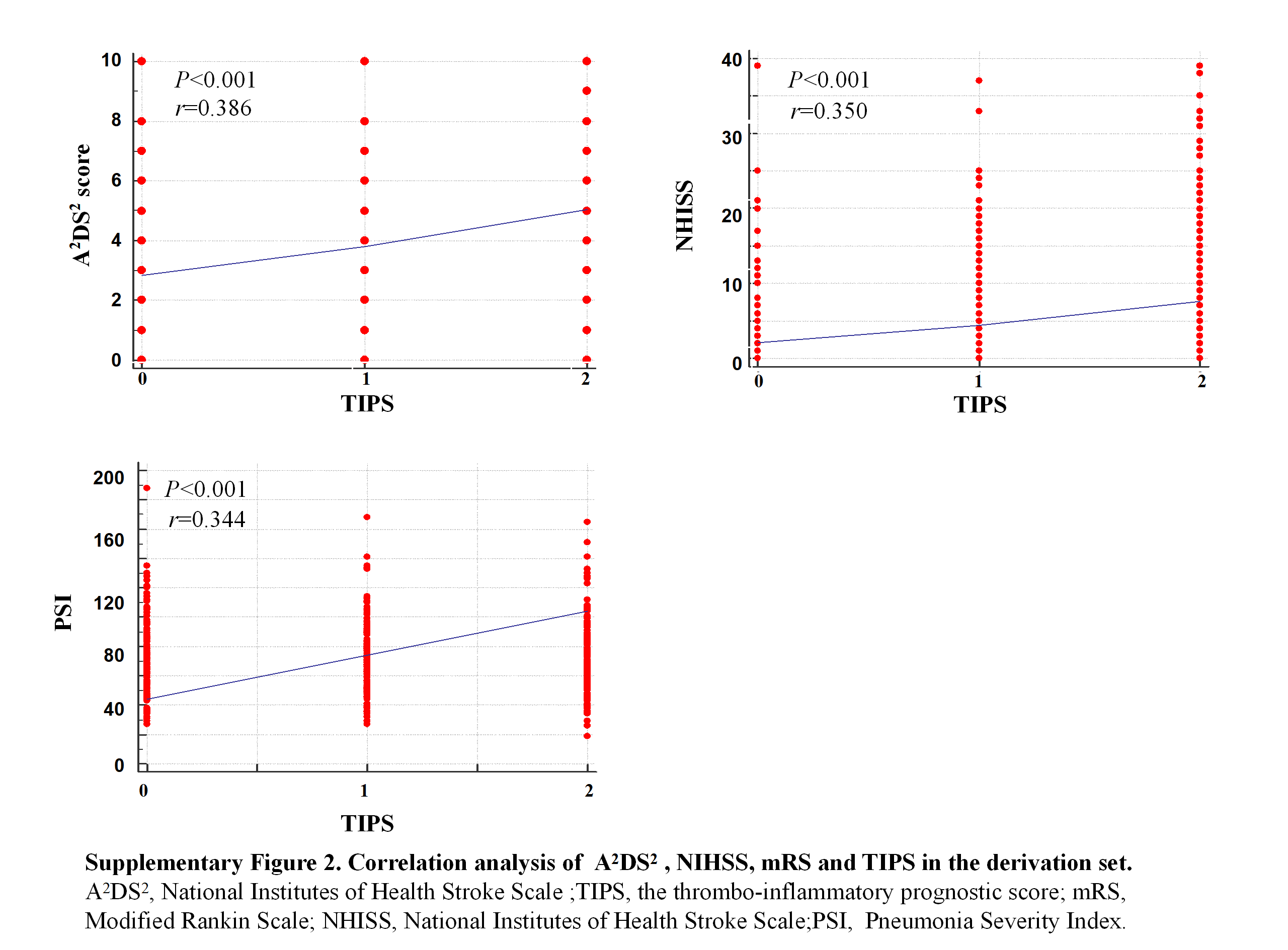


Supplementary Figure 2. Correlation analysis of A^2^DS^2^, NIHSS, mRS and TIPS in the derivation set.

A^2^DS^2^, National Institutes of Health Stroke Scale ;TIPS, the thrombo-inflammatory prognostic score; mRS, Modified Rankin Scale; NHISS, National Institutes of Health Stroke Scale;PSI, Pneumonia Severity Index.


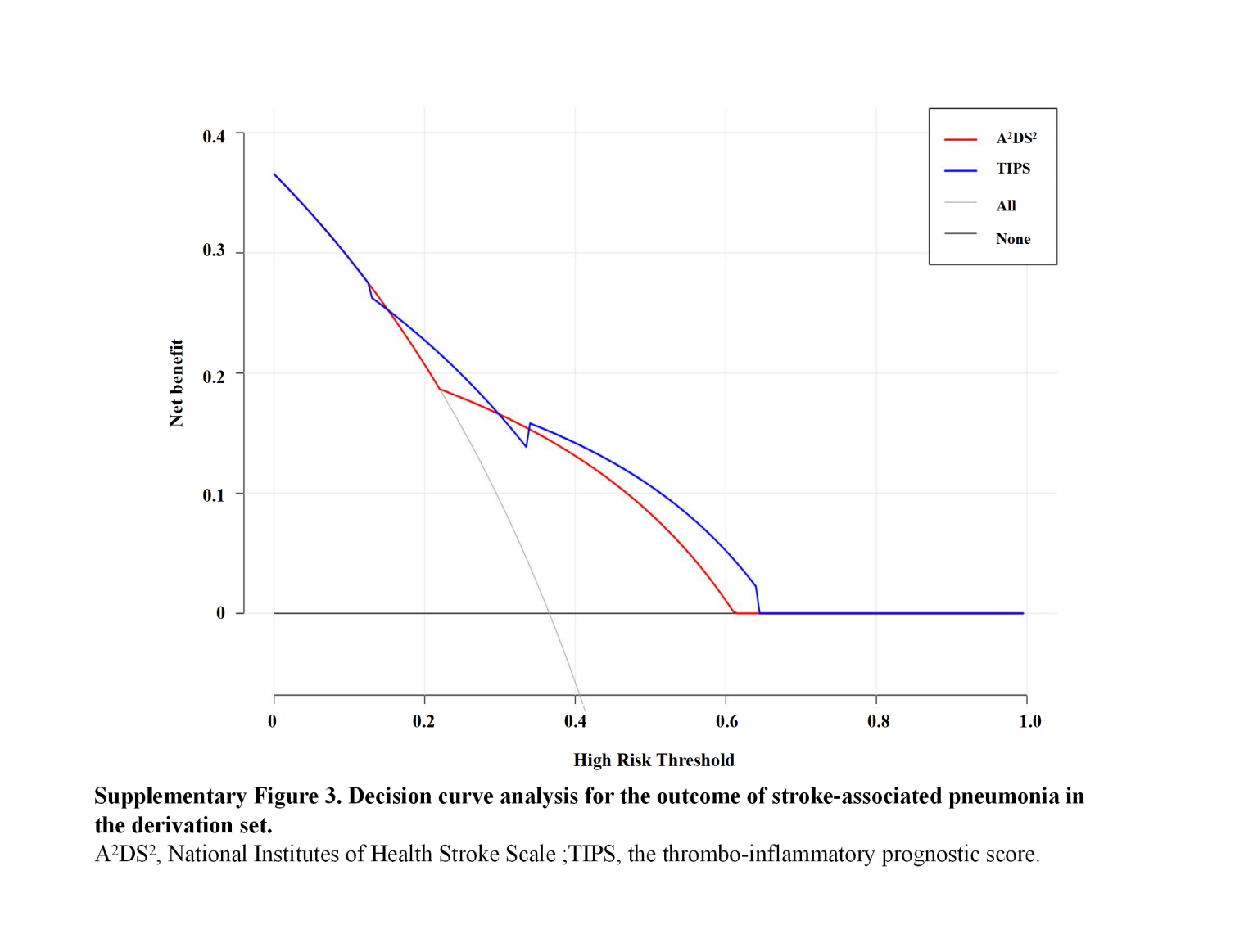


Supplementary Figure 3. Decision curve analysis for the outcome of stroke-associated pneumonia in the derivation set.

A^2^DS^2^, National Institutes of Health Stroke Scale ; TIPS, the thrombo-inflammatory prognostic score.


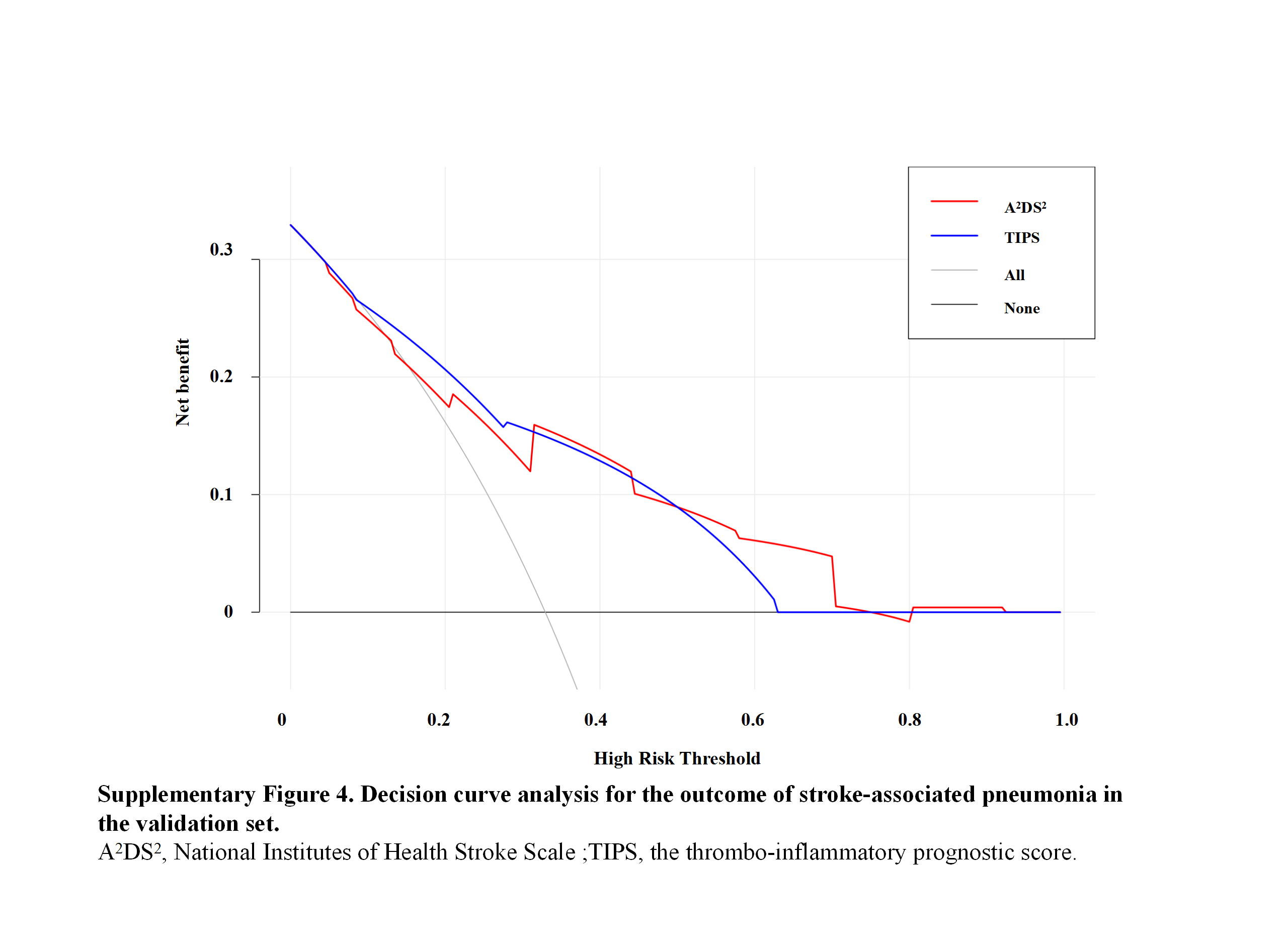


Supplementary Figure 4. Decision curve analysis for the outcome of stroke-associated pneumonia in the validation set.

A^2^DS^2^, National Institutes of Health Stroke Scale; TIPS, the thrombo-inflammatory prognostic score.
